# A psychological experience assessment protocol of parent caregivers in paediatric palliative care

**DOI:** 10.1080/07853890.2023.2268093

**Published:** 2023-11-27

**Authors:** Alexandra Jóni Nogueira, Maria Teresa Ribeiro

**Affiliations:** CICPSI, Faculty of Psychology, University of Lisbon, Alameda da Universidade, Lisboa, Portugal

**Keywords:** Paediatric palliative care, life-limiting conditions, parent caregivers, family, psychological assessment, qualitative methods

## Abstract

**Background:** Paediatric Palliative Care (PPC) has undergone rapid growth in Portugal, where there are over 7800 children with life-limiting conditions. This is a complex experience for families due to the ongoing threat and vulnerability caused by the emergence of an illness, and therefore several studies have tended to focus on the adaptation of parent caregivers. The aim of the present study is to present a psychological experience assessment protocol of parents in PPC. **Methods:** It consists of a socio-demographic and clinical questionnaire and a semi-structured interview based on an incomplete narrative deriving from the *Unwanted Guest Metaphor*. **Results:** On the basis of the latter, 10 dimensions of the experience in the parental subsystem were explored through parents’ own perspective, namely: confrontation with the diagnosis; representation of the illness; emotional impact; day-to-day challenges; family impact; resources and social support; coping strategies; posttraumatic growth; representation of the sick child; and future perspectives. **Discussion:** The protocol can be used in person or remotely and its application enables the identification of specific needs and the establishment of psychotherapeutic goals and strategies for each family, thus enhancing their well-being and resilience, from an eco-systemic perspective. **Conclusions: **The protocol is presented in detail and its importance in the context of research and systemic intervention in PPC is discussed.

## Background

Paediatric palliative care has been recognized as a basic human right by the World Health Organization since 1988 and is currently a developing area. This care seeks to promote the physical, psychological, and spiritual well-being of the sick child/youth, but also of all his/her relatives, resulting in the improved quality of life of all the participants in this context [1]. According to [2], this care is holistic and proactive and is intended for children and young people with complex chronic illnesses [3] from which they will not get better.

To facilitate the identification of children with palliative needs, a set of categories was constructed in which each illness can be included depending on its characteristics [4]: Category I is related to life-threatening conditions for which curative treatment may be feasible but can fail (e.g. cancer, transplant, and children on long-term ventilation); Category II refers to conditions where premature death is inevitable, but can involve long periods of intensive treatment and allow for participation in daily activities (e.g. cystic fibrosis, Duchenne muscular dystrophy); Category III is for progressive conditions without curative treatment options, where treatment is exclusively palliative and may commonly extend over many years (e.g. iedsevere cerebral palsy, complex disabilities).

According to data from 2018, an estimated 7828 Portuguese children/youth require palliative care [5]. Portugal has evolved rapidly and prominently in Europe in the provision of this care and has been positioned at Level Four since 2018 in view of its ample provision of palliative care for children with training and targeted plans for service development and integration in the health services [6]. Broader provision of paediatric palliative care has also been seen on a worldwide scale. Countries such as Australia, Germany, the United Kingdom and the United States of America are currently at Level Five, which means that they have a comprehensive provision of palliative care for children, with a fully integrated approach to health services, as well as national policies supporting children’s palliative care.

The onset of a potentially fatal disease in the paediatric age is a stressor which has a profound impact on the sick child/youth, but also on the whole family, with implications at the level of its structure and dynamics, thus calling for systemic reorganization [7]. This is particularly important because families and healthcare professionals have tended to invest more in the family over the hospital setting, and therefore most children with palliative needs live at home [8]. This means that the child/youth’s household lives with their illness and is involved in their care on a daily basis, making it essential to understand the impact of the illness on the various members of the family system - parent caregivers, healthy siblings, grandparents and other significant people in each family.

The set of emotions, thoughts and behaviours stemming from and/or experienced within the context of an illness is referred to as a psychological experience. Thus, by promoting a reflection on the psychological experience of the different family members in the context of an illness [9], it is possible to gain further awareness of their own needs and align them with the intervention provided by the multidisciplinary health team [10]. To this end, a literature review along with the development of protocols for the assessment of the psychological experience of each family subsystem, namely mothers and fathers is necessary.

## Parent caregivers

Parental stress has been widely studied in this context of life-limiting illness (e.g. [11,12]). The moment of diagnosis of the illness [13], the continuous caregiving, the need for hospitalizations, and readmissions to health care services are associated with higher stress in the parent caregivers [14]. According to [15], the age of the sick child/youth and the caregivers’ level of schooling are important predictors of the psychological distress of the parental subsystem. Otherwise, parent caregivers also tend to experience anxiety and fear caused by the possibility of losing their child and owing to the ambiguity resulting from the uncertainty and anguish of decision-making, especially end-of-life decisions [16,17].

In the study conducted by [18] with 264 parent-caregivers of children with cancer in Jordan, 75.4% of them were found to manifest mild to severe levels of burden. Low socioeconomic resources, significant symptomatology of depression and/or anxiety and the child/youth having symptoms such as pain are predictors of a greater burden [19] also mention that the caregivers’ characteristics, the family system, the available health resources and services and the sociocultural context itself directly impact the perception of burden.

It is therefore essential to consider the family and social network of the caregivers and, above all, dyadic coping in the parental subsystem [20], which directly affects co-parenting and conjugality [21,22] corroborate these findings, concluding that compared to parents of healthy children, parents of children with palliative needs have a lower quality of life, high levels of burden and significant psychological distress. The study by [23] also concludes that quality of life is impacted in different dimensions. Parents report a constant feeling of fatigue and stress and worry about the future. Additionally, they deprive themselves of leisure and socialization opportunities, are confronted with financial difficulties, and have to manage society’s value judgments regarding disability. Due to the time invested in caring for the sick child/youth, parents minimize their attention to their healthy children, which gives rise to feelings of guilt and sadness.

Several empirical studies have pointed to a tendency for mental disorders following the experience of a potentially traumatic event, such as the confrontation with a life-limiting condition e.g. [13]. However, other research also shows that it is possible to react actively to traumatic situations and that people can learn and identify benefits from them, allowing them to continue to live and be functional [24], which is called posttraumatic growth [25]. For instance, [26] study concludes that over half of the participant parents of children with special health needs manifested changes in life appreciation and personal growth and 39% changes in the spiritual and religion dimension.

When asked about their needs, parents emphasize the importance of receiving emotional support, more knowledge about the illness, practical day-to-day support, having time to themselves, gaining more control over the future and knowing who they can count on, e.g. [22,27,28] highlight the pertinent role of paediatric palliative care services in ensuring that families’ mental health is assessed appropriately and regularly. In fact, counselling psychology can be applied in this context aiming to promote well-being and in order to meet these needs. It can be essential to promote social support [29], and emotional expression, to foster optimism and hope (e.g. [30]), as well as create the opportunity to share their experience and story to feel understood and supported [31]. In order to promote these psychological interventions, it is essential to create structured family assessment protocols.

## The importance of psychological assessment

Psychological assessment, in its scientific domain, is a disciplinary area that encompasses the development, construction, and evaluation of the most appropriate means of obtaining and analyzing information about a person (e.g. [32]). A comprehensive psychological assessment includes the different members of the family system, along with the relational dynamics among them, the extended family, and the contexts in which they act and influence each other [10,33]. This assessment occurs in the affective, cognitive, structural, behavioural, and communication domains, using various techniques (e.g. [34]).

The construction of methodologies to guide the process of psychological assessment in different circumstances and specifically for families in the context of paediatric palliative care is considered necessary and pertinent. The literature reinforces the importance of specific protocols for the various family subsystems, namely for the parental subsystem. Research is mostly quantitative in nature, with protocols that include psychological assessment instruments for previously defined dimensions, such as quality of life, parental stress, caregiver burden, and family functioning. In addition, there are some quantitative tools that are used to assess parent caregivers in this context as Quality of Life During Serious Illness – Family Carers (QOLLTI-F) [35], Family Appraisal of Caregiving Questionnaire for Palliative Care (FACQ-PC) [36] and Coping Health Inventory for Parents (CHIP) [37]. Furthermore, there are several studies of mixed methodology and only qualitative, whose protocols are based on semi-structured interview scripts.

This protocol for assessing the experience of parent caregivers of children with a complex chronic illness should be used for research purposes - aiming to increase knowledge and contributions that allow for a better understanding of the specific experiences of this population - but also for clinical purposes, enabling its effective and useful implementation by psychologists who work in this context for their psychological intervention. Based on its application, it is possible to collect the necessary information on the different dimensions of the psychological experience of this family subsystem in a more comprehensive, targeted, and personalized manner. These contributions are central to the establishment of objectives and interventional strategies with each family in the context of paediatric palliative care, leading to the promotion of guidelines that foster further development of their well-being, coping and family resilience in a holistic and dynamic approach.

On the other hand, it offers the researcher and/or psychologist the opportunity to use a structured and scientifically grounded assessment methodology, where the mother or father caregiver may share his/her own perspective and deepen his/her narrative in a flexible manner. It is therefore essential that the assessment protocol be based on qualitative methodologies, which are considered the best means to explain the processes and phenomena of specific contexts, where the use of semi-structured interviews is particularly relevant [38].

In addition, to date, most of the literature has focused on oncological diseases, giving less attention to neurological, renal, cardiovascular, congenital and haematological diseases, among others. Therefore, it is important to develop an assessment methodology that allows for the collection of information in multiple disease contexts within the scope of paediatric palliative care. Hence, this research aims to contribute to the structuring of a protocol for the assessment of the experience of the parental caregiver subsystem, considering the heterogeneity of existing complex chronic diseases, thus filling two gaps in scientific research.

## Methods

### Design and variables

This study uses a qualitative and exploratory design and was approved by the *Comissão Especializada de Deontologia do Conselho Científico* [Board of the Ethical and Deontological Committee] of the Faculty of Psychology, University of Lisbon.

Based on the above considerations, this study presents a methodology for assessing the psychological experience of parent caregivers of children/youths with a complex chronic illness. Through its implementation, it seeks to contribute to an understanding of the psychological experience of the illness from their own perspective, which is essential in a population considered at-risk [39].

Upon completion of the literature review, the objective was established to explore the following themes: confrontation with the diagnosis; representation of the illness; emotional impact; day-to-day challenges; family impact; resources and social support; coping strategies; posttraumatic growth; representation of the sick child; and future perspectives.

This protocol makes it possible to explore the specific features of parents’ psychological experience, resulting in the identification of specific needs and resources, in a holistic and eco systemic approach. Finally, it promotes the development of skills and the activation of fundamental processes for the elaboration of family resilience and posttraumatic growth in this population.

### Patient selection

The assessment protocol is intended for mother and father caregivers of children/youths with a complex chronic illness - falling into one of the four groups identified in the literature [31] - that was diagnosed at least 12 months ago. Participants must be 18 years of age or older and live with the child/youth requiring paediatric palliative care. The child/youth must be 18 years of age or younger. It should be noted that participation is individual and the parent caregivers must be able to read and write in English unless the psychologist/researcher can do so for the participant.

### Data collection

In order to construct the semi-structured interview script, the parent caregivers’ psychological experience of the particularities of the illness was initially identified and characterized, anchored on an extensive and updated literature review. Subsequently, these dimensions served as the basis for the construction of questions that facilitated the collection of the desired information in an effective and relaxed manner, due to the implicit creativity of each question and the relief resulting from the opportunity of exposure through a previously organized and contextualized narrative. The questions were organized in a sequential and structured manner, based on an incomplete narrative that emerged from the *Unwanted Guest* metaphor [40], which is presented as the unwanted appearance of the child/youth’s complex chronic illness in the life of the entire family system.

This script was elaborated on the basis of Narrative Therapy strategies [41], promoting a conversational interaction between the participant and the researcher/psychologist. Thus, the content of each participant’s answers reflects their dominant narrative about the way they experience their child’s illness [42].

To characterize the parent caregivers and their personal and family circumstances, a socio demographic and clinical data sheet was also designed, where initial information on the caregivers themselves, the sick child/youth and their family was collected.

The structured clinical assessment tool of the parent caregivers’ psychological experience of the illness is thus composed of a socio-demographic and clinical data sheet and a semi-structured interview script, to which an informed consent form was added to ensure compliance with ethical aspects.

### Analysis

The responses resulting from this protocol, namely the script, should be understood using a reflexive thematic analysis of the narrative, through an inductive-deductive process [43,44]. This analysis is widely used within health and wellbeing research, and it is a useful qualitative approach for more applied research [45]. In counselling and psychotherapy studies, reflexive thematic analysis has been recognized as a flexible and accessible method [46].

The practices of coding and theme development, the possibility of capturing semantic and/or latent meaning and orienting to data inductively and/or deductively are the key features. In fact, this qualitative analytic approach requires depth of engagement, thinking creatively and reflexively about the data and a coding process designed to parse out different facets of data meaning and not only the most superficial information [47]. Nevertheless, it is important to emphasize that should not be thought of as an atheoretical analysis [46].

Despite this, the choice of method for analyzing the information collected is up to the professional applying this protocol, depending on the clinical or research aims for which it is intended, and for which reflexive thematic analysis may not be the most appropriate [48].

## Results

Based on the above considerations, the assessment protocol is presented in detail.

### Socio demographic and clinical data sheet

This sheet aims to collect initial information that characterizes the participant and his/her family life, namely through data regarding the parent caregivers themselves, the child/youth with the complex chronic illness and the family (e.g. [49]).

#### Data related to the father/mother caregiver

Information regarding the following variables is requested: family relationship, age, gender, nationality, marital status, district of residence, education, occupation, employment status and past and present use of psychiatric/mental health services.

#### Data on the child/youth with life-limiting conditions

Information is collected on: age, gender, illness diagnosis, time elapsed since diagnosis, technological dependence, hospitalizations (number and last time), level of disability of the child/youth, institutional support, primary caregiver and secondary caregivers (if any) and knowledge regarding paediatric palliative care.

#### Family data

The aim of collecting this data is to gain additional knowledge about the family’s circumstances, namely: household (number of members, apart from the participant and the sick child/youth) and characterization of all the members (according to gender, age, school attendance and/or professional occupation).

### Semi-structured interview script

This script is based on an incomplete narrative based on the *Unwanted Guest* Metaphor - also referred to as *Joe-the-Bum Metaphor*, used in Acceptance and Commitment Therapy -, where the guest is understood as the child’s illness. The main purpose of using this metaphor is to gain further awareness of the participant’s stance on the thoughts, emotions and memories that develop in this context. Therefore, it seeks to favour perceived control over the illness by placing some limits on it [40].

The narrative presented in this script includes 15 interspersed questions that refer to the 10 dimensions under assessment. These dimensions correspond to the particularities of the illness adaptation process in this population and were chosen upon completion of the literature review, namely: confrontation with the diagnosis; representation of the illness; emotional impact; day-to-day challenges; family impact; resources and social support; *coping* strategies; posttraumatic growth; representation of the sick child; and future perspectives. Each question, or set of questions, aims to operationalize each of the afore-mentioned domains. The script proposed within this methodology for assessing parent caregivers’ psychological experience of illness is presented in its entirety below.

*Imagine today was just like one of your average days before your child’s illness was diagnosed. Recall how you felt, what your routine was and what your home was like at that time, together with your family.*

*Now imagine that at that very moment an ‘unwelcome guest’ enters your home. This ‘guest’ has suddenly appeared and is not welcome, because it is your child’s illness.*

**What colour would best represent this moment for you? Please justify your choice.** (Confrontation with the diagnosis)

*From this moment on, this ‘unwelcome guest’ begins to live more closely with you and your family. You are already able to recognize him and know more about him.*

**If your child’s illness had an identity card, which three characteristics would you choose to define it?** (Representation of the illness)

*Despite daily coexistence with this ‘guest’, its presence has triggered various worries, emotions and feelings. Many people in the same circumstances report feeling distressed, afraid, anxious and angry. There are also family members who manage to find some joy.*

**If what you feel could form a puzzle with different sized pieces, what emotions would form the larger pieces?** (Emotional impact)

*From this moment on, you start trying to expel this ‘unwanted guest’ from your life and from your family. Your efforts focus on trying to maintain some normality in your daily life in the face of the many challenges that this ‘guest,’ which is the illness, has brought you.*

**If someone could have alerted you to the main challenges you are currently experiencing, what would you have liked them to tell you?** (Day-to-day challenges)

**If you were to switch places with the parents of a totally healthy child right now, what do you think they would feel in your place that they do not feel on a day-to-day basis?** (Day-to-day challenges)

**On a scale of one to ten, how overburdened do you currently feel?** (Day-to-day challenges)

*In addition to the impact that the presence of this ‘guest’ has had on you, it is likely to have also had an impact on your entire family.*

**When you think about how your family relates to each other, how they communicate and how they support each other, what do you consider to be the main changes that have occurred since the illness?** (Family impact)

**If I asked your child’s illness to tell me about you, what do you think she would tell me?** (Family impact)

**What would you like to say to this ‘unwanted guest’?** (Family impact)

*At some point, you realize that this ‘guest’ has settled in and does not seem to be going anywhere. At this point, it is important to gain focus and identify the resources that are available to you. Think about your family, friends, neighbours, health care professionals and other people who may be important to you.*

**If you could give three awards to the people who have helped you the most, who would you give them to and why?** (Resources and social support)

*This unwelcome ‘guest’ has already been in your life for some time and so far, you have had to find different ways of dealing with this situation. However, there is another family that has just received the diagnosis of their child’s illness and does not know how to react or what to do.*

**If you could talk to them and tell them three of your ‘tricks’, to help them cope better with the illness, what would you tell them?** (*Coping* strategies)

*By coexisting on a daily basis and for so long with this ‘unwanted guest,’ you begin to realise that something may have changed in you or in your family. But this time it has changed for the better. The presence of this ‘guest’ is still unwanted, but it no longer seems to bother you as much as it did at the beginning.*

**What interesting discoveries have you made about yourself and your family?** (*Posttraumatic growth*)

*Very good. Imagine now that someone has the power to change the available resources and create a support network that could help your family cope better with this illness situation.*

*You happen to meet this person in the elevator of a building and you have around 30 s to tell him/her your child’s story.*

**What would you tell him/her?** (Representation of the sick child)

*This person was most interested to hear your story and, before you leave the elevator, asks you about your future.*

**If your family history was told in a book, what would be written on the last page?** (Future perspectives)

*Finally, imagine that this person asks you for three ideas that you think are necessary, important and useful for you and all families in the same circumstances.*

**What ideas would you give him?** (Future perspectives)

*Your interview has come to an end. Thank you very much for your participation in this study and for your commitment to cope better with the ‘unwanted guest’ who has entered your family.*

## Discussion

The dimensions mentioned in the theoretical framework - which correspond to the specific features of parent caregivers’ psychological experience - and which are explored through the application of this protocol, also aim to represent psychological intervention themes proposed for this population. In other words, the answer to each question (or set of questions) regarding a dimension seeks to obtain information which will prove to be useful and necessary for an in-depth understanding of the psychological experience. This will lead to an identification of concrete needs, as well as strategies, resources and competencies that may benefit from development or mobilization. The final goal is to further promote the illness adaptation process, as well as the family’s well-being and resilience [24]. The information from psychological assessment can also assist other health professionals in the multidisciplinary team, in addition to psychologists.

This protocol can be applied in person or remotely and the participants’ privacy should be ensured in both contexts. In addition, this assessment protocol can be applied more than once to the same participant if it is properly phased in time, according to the research or psychotherapeutic goals of its application. This strategy provides an opportunity to explore any changes in the narrative over time, since the construction of an alternative narrative may be the result of a process of co-construction experienced within the scope of psychological intervention [42].

It should also be noted that the very application of the protocol can translate into a therapeutic effect for parent caregivers, as it represents an opportunity to create stories and keep memories [9]. In addition, the narrative written in the script emphasizes that there are other parents in identical circumstances, which can also promote some relief and peer identification [31]. The possibility of emotional expression [28] and the safe and unbiased relationship established between the participants and the psychologist/researcher are also highlighted.

Nevertheless, although psychological assessment in the health context plays an important role in understanding psychological phenomena, there are some limitations that should be considered. First, there is no universal model of the psychological experience of parent caregivers and which of its dimensions are considered more important and/or necessary to assess. Although this assessment protocol attempts to integrate the various particularities of the adaptation to illness in this population, there may be others that could also be incorporated into the assessment. Second, there is an inherent limitation to any psychological assessment process, which is the probabilistic nature of its conclusions. Although it is important to consider this factor, this protocol should be used in clinical practice, with the construction of a therapeutic relationship of trust and proximity and with the implicit flexibility and balance of a psychological intervention.

## Conclusions

Despite the existence of general assessment parameter guidelines, there is a clear lack of empirically grounded theoretical and methodological frameworks specifically geared towards assessing the experience of parent caregivers. It is in this context that the rationale for an assessment process within the scope of paediatric palliative care and for the parental subsystem emerges, with a view to standardizing the collection of information and defining key dimensions for understanding and analysis. The present structured clinical assessment tool results from an attempt to respond to the clinical and research needs of professionals. It advances important information for the development of a psychotherapeutic intervention tailored to the characteristics, needs and resources of each parental caregiver, considering their personal, family and socio-cultural circumstances in the context of the illness.

This assessment protocol is flexible, and it can be used in person or remotely and alone, in its entirety, or combined with other psychological assessment instruments, due to the diversity of information it allows to collect. The researcher/psychologist should guide the application of this protocol towards the dimensions that prove to be most useful and/or necessary in each family. Therefore, the presented assessment tool may contribute to the scientific development of the psychological assessment of parents in the context of paediatric palliative care, and its application is recommended in studies in this field and in clinical practice, with families in these circumstances.

## Data Availability

Data is contained within the article.
